# An *in vitro* method for detecting genetic toxicity based on inhibition of RNA synthesis by DNA lesions

**DOI:** 10.1186/s41021-015-0014-8

**Published:** 2015-08-01

**Authors:** Yuina Sonohara, Shigenori Iwai, Isao Kuraoka

**Affiliations:** Division of Chemistry, Graduate School of Engineering Science, Osaka University, 1-3 Machikaneyama, Toyonaka, Osaka 560-8531 Japan

**Keywords:** DNA lesion, Inhibition of RNA synthesis, Genetic toxicity assay

## Abstract

**Introduction:**

A wide variety of DNA lesions such as ultraviolet light-induced photoproducts and chemically induced bulky adducts and crosslinks (intrastrand and interstrand) interfere with replication and lead to mutations and cell death. In the human body, these damages may cause cancer, inborn diseases, and aging. So far, mutation-related actions of DNA polymerases during replication have been intensively studied. However, DNA lesions also block RNA synthesis, making the detection of their effects on transcription equally important for chemical safety assessment. Previously, we established an *in vivo* method for detecting DNA damage induced by ultraviolet light and/or chemicals via inhibition of RNA polymerase by visualizing transcription.

**Results:**

Here, we present an *in vitro* method for detecting the effects of chemically induced DNA lesions using *in vitro* transcription with T7 RNA polymerase and real-time reverse transcription polymerase chain reaction (PCR) based on inhibition of *in vitro* RNA synthesis. Conventional PCR and real-time reverse transcription PCR without *in vitro* transcription can detect DNA lesions such as complicated cisplatin DNA adducts but not UV-induced lesions. We found that only this combination of *in vitro* transcription and real-time reverse transcription PCR can detect both cisplatin- and UV-induced DNA lesions that interfere with transcription.

**Conclusions:**

We anticipate that this method will be useful for estimating the potential transcriptional toxicity of chemicals in terminally differentiated cells engaged in active transcription and translation but not in replication.

## Introduction

Genomic DNA is continuously damaged by various exogenous and endogenous agents [1, 2]. The induced DNA lesions interfere with replication, leading to mutations and cell death, and have been associated with cancer, inborn diseases, and aging. In addition, DNA lesions interfere with transcription, inhibiting elongation by RNA polymerase and leading to reduced transcription and/or mutations of the transcript [3, 4]. Therefore, DNA lesions can induce cytotoxicity by inhibiting replication and/or transcription.

Under laboratory conditions, cell lines are maintained in an environment favorable to growth, DNA repair, prevention of apoptosis, and other aspects of cellular metabolism [3, 5–7]. As cancer and stem cells divide rapidly and constantly, proper experimental conditions for such cell lines are focused on replication. However, most cells within the human body are not exposed to such experimental conditions and are terminally differentiated, and post-mitotic cells engage in active transcription and translation but not in replication. Hence, transcription is assumed to be as important as replication for estimating the genetic toxicity of DNA lesions in human organs.

Inhibition of transcription can be effected by RNA polymerase stalling due to DNA lesions or RNA polymerase II inhibitors such as alpha-amanitin from the death cap [8, 9] and actinomycin D from *Streptomyces* bacteria, which intercalate into DNA and activate an inducer of apoptosis in many cell lines [10, 11].

All known organisms have repair systems to remove DNA lesions and maintain genomic integrity. When RNA polymerase II encounters DNA damage that blocks transcription during the elongation phase, transcription-coupled repair (TCR) immediately counteracts the interference. Previous studies have demonstrated TCR responses to bulky and helix-distorting lesions such as ultraviolet light (UV)-induced photolesions, e.g., cyclobutane pyrimidine dimers (CPD) [12, 13] and 6–4 photoproducts (6–4 pp) [14], cisplatin intrastrand crosslinks [15], and benzopyrene adducts [16].

Assessment of the biological risk and toxicity of newly synthesized chemicals is hampered by the large number of substances and the complexities of living cells. Current methods for detecting toxic substances are based on the DNA damage response in replication and cell proliferation. For example, the micronucleus assay that detects small nuclei is a well-established method for monitoring genetic toxicity of test substances in the environment [17], and the comet assay (single-cell gel electrophoresis) is a simple and sensitive method for measuring chemically induced DNA damage [18, 19]. Despite the availability of numerous assays to detect genotoxic chemicals, there was no simple method to estimate their effects on transcription until recently.

In our previous work, we established a method for detecting the effects of chemically induced DNA damage on transcription using the nucleotide analog 5-bromouridine and a anti-5-bromouridine antibody and/or the nucleotide analog 5-ethynyluridine [20] and a click chemistry reaction [21, 22] without radioisotopes [23]. Our method is based on a model for transcription-coupled nucleotide excision repair (NER) triggered by blocked transcription at DNA lesions [24, 25]. The method employs common human cells without genetic modifications and terminally differentiated PC12 cells that actively undergo transcription but not replication, and it can detect a wide range of DNA lesions within 8 h of exposure to UV and/or chemicals, e.g., camptothecin, etoposide, 4-nitroquinoline 1-oxide (4NQO), mitomycin, and cisplatin.

Here we present an *in vitro* assay for detecting the effects of chemically induced DNA lesions using *in vitro* transcription and real-time reverse transcription polymerase chain reaction (PCR). The method is based on the inhibition of *in vitro* RNA synthesis for chemically treated DNA templates and allows evaluation of the potential effects of chemicals on transcription in terminally differentiated human cells.

## Materials and methods

### Enzymes and chemicals

T7 RNA polymerase (T7 RNAP) and reverse transcriptase were purchased from TOYOBO (Osaka, Japan). RNase inhibitor was from Wako (Osaka, Japan), restriction enzymes were from New England Biolabs (Ipswich, MA, USA), Fast SYBR Green Master Mix was from Life Technologies (Carlsbad, CA, USA), and cisplatin was from Sigma-Aldrich (St. Louis, MO, USA).

### DNA treatment

Plasmid (pBluescript II SK (−) containing the T7 promoter; Stratagene, La Jolla, CA, USA) DNA templates were purified using the QIAGEN Midi Kit (QIAGEN, Hilden, Germany). For UV irradiation of DNA templates, UV-light (254 nm, 450 J/m^2^) was used. For cisplatin treatment, DNA templates were incubated with cisplatin (drug/nucleotide ratio = 0.005) at 37 °C overnight.

### Polymerase chain reaction (PCR)

For PCR, UV-irradiated or cisplatin-treated DNA samples were used with primers 2140–2159 (5′-tatcagcaataaaccagcca-3′) and 2440–2421 (5′-gcggccaacttacttctgac-3′) and EmeraldAmp PCR Master Mix (TaKaRa, Otsu, Shiga, Japan) according to the manufacturer’s instructions. PCR products were analyzed by 1 % agarose gel electrophoresis.

### *In vitro* transcription

For *in vitro* transcription, 50-μL reactions containing 100 ng DNA template, 4 mM NTP mixture (ATP, CTP, GTP, and UTP), and 5 units thermo T7 RNAP in buffer (40 mM Tris–HCl, pH 8.0, 50 mM NaCl, 8 mM MgCl_2_, 5 mM dithiothreitol, 20 units RNase inhibitor) were incubated at 37 °C for 1 h. RNA transcripts were purified using an RNeasy Mini Kit (QIAGEN) with RNase-Free DNase (QIAGEN) according to the manufacturer’s instructions. Transcription products were analyzed by 1 % agarose gel electrophoresis.

### Reverse transcription (RT)-PCR

cDNAs were generated from purified RNA samples using primer 2440–2421 (5′-gcggccaacttacttctgac-3′) and ReverTra Ace reverse transcriptase (TOYOBO) according to the manufacturer’s instructions.

### Real-time quantitative PCR (qPCR)

Real-time quantitative PCR (qPCR) was performed on a StepOne System (Life Technologies) using Fast SYBR Green Master Mix (Life Technologies) with primers 2140–2159 (5′-tatcagcaataaaccagcca-3′) and 2440–2421 (5′-gcggccaacttacttctgac-3′) to ensure the appearance of a single product peak (301 bp) from mock mixtures in the melting curve analysis. Each reaction was run in triplicate, and the data were plotted as ∆Rn versus cycle number.

## Results and discussion

**Fig. 1:**
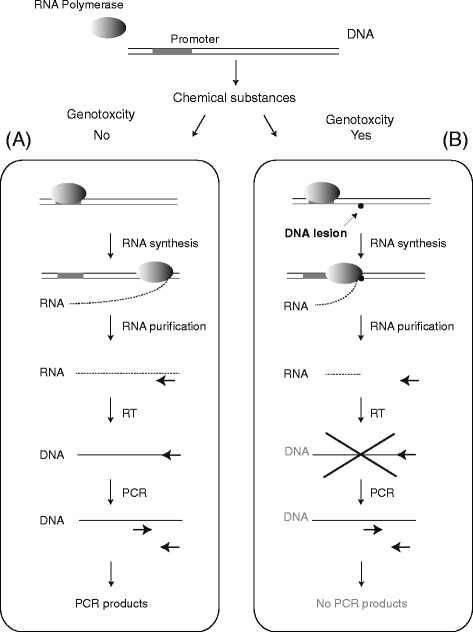
Experimental design. **a** In the absence of DNA damage, RNA polymerase generates RNA transcripts from DNA templates (*normal transcription*). After purifying RNA, RT-PCR is performed, and the PCR products are analyzed. **b** If chemical substances damage DNA, the resulting lesions inhibit RNA synthesis, as RNA polymerase cannot synthesize transcripts from damaged templates, and PCR products will not be detected

**Fig. 2:**
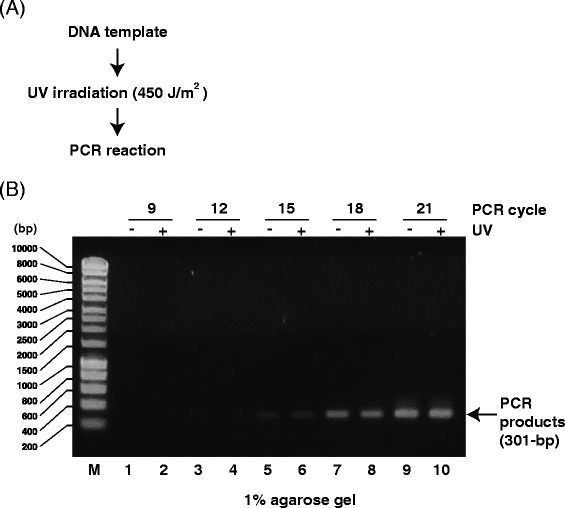
Analysis of PCR products from UV-irradiated DNA templates. **a** Scheme for detecting DNA damage using PCR products from UV-irradiated (450 J/m^2^) DNA templates. **b** Agarose gel (1 %) demonstrating PCR products (301 bp) from UV-irradiated DNA templates. M: size marker. Odd lanes: PCR products from mock DNA templates. Even lanes: PCR products from UV-irradiated DNA templates. DNA was amplified for 9 (*lanes 1 and 2*), 12 (*lanes 3 and 4*), 15 (*lanes 5 and 6*), 18 (*lanes 7 and 8*), or 21 (*lanes 9 and 10*) PCR cycles

Previously, we demonstrated that our *in vivo* method for visualizing transcription in mammalian cells can detect UV- and/or chemically (e.g., camptothecin, etoposide, 4NQO, cisplatin) induced damage in genomic DNA by inhibiting RNA polymerase during transcription elongation [20, 22, 23]. Here, we modified this *in vivo* method to establish a new *in vitro* method (Fig. 1) using T7 RNAP and DNA templates containing a T7 promoter. RNA polymerase synthesizes new transcripts from a DNA template, which can be detected by RT-PCR (Fig. 1a). However, RNA polymerase stops at chemically induced DNA lesions, and no products are detected by RT-PCR (Fig. 1b). First, we tested the detection of UV-induced DNA lesions (e.g., CPD and 6–4 pp that trigger NER) using conventional PCR. Under these experimental conditions, approximately 12 DNA lesions were expected in plasmid DNA [26]. Although 6–4 pp is more frequently induced than CPD, both lesions are thought to inhibit mammalian RNA polymerase II transcription almost completely [14, 27]. However, conventional PCR (Fig. 2a) could obscure the difference between UV-irradiated and non-irradiated DNA (Fig. 2b), as Taq DNA polymerase might synthesize DNA from the undamaged region (301 bp) in the UV-irradiated DNA template and/or beyond UV lesions such as CPD or 6–4 pp in the PCR cycles [28]. In contrast, T7 RNAP covers the DNA sequence between the T7 promoter and the PCR-detecting region and shows inefficient RNA primer extension from arrested RNAs in a single reaction [29]. This approach seems to be more suitable for detecting DNA lesions than conventional PCR as it reveals the effects of DNA lesions on transcription.

**Fig. 3:**
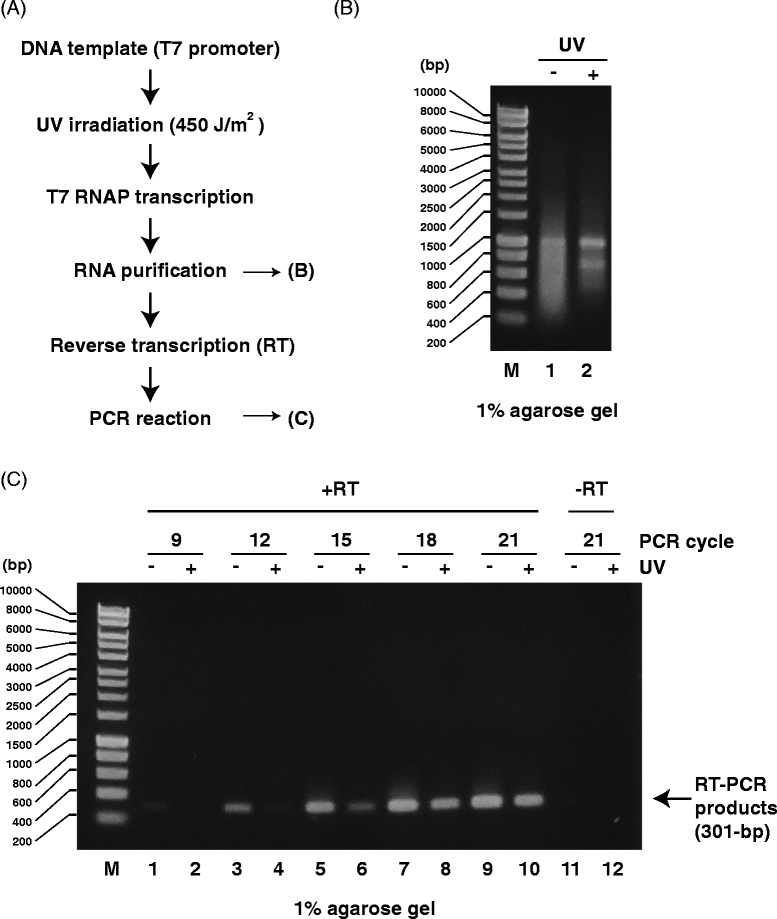
Analysis of PCR products generated by RT-PCR from RNA transcripts of UV-irradiated DNA templates. **a** Scheme for detecting transcription inhibition using RNA transcripts from UV-irradiated (450 J/m^2^) DNA templates. **b** Agarose gel (1 %) demonstrating RNA transcripts from UV-irradiated DNA templates. M: size marker. Lane 1: RNA transcripts from mock DNA templates. Lane 2: RNA transcripts from UV-irradiated DNA templates. **c** Agarose gel (1 %) demonstrating RT-PCR products (301 bp) from UV-irradiated DNA templates. M: size marker. Odd lanes: RT-PCR products from mock DNA templates. Even lanes: RT-PCR products from UV-irradiated DNA templates. DNA was amplified for 9 (*lanes 1 and 2*), 12 (*lanes 3 and 4*), 15 (*lanes 5 and 6*), 18 (*lanes 7 and 8*), or 21 (*lanes 9 and 10*) PCR cycles. Lanes 11 and 12: no RT reaction

When we tested this method to detect transcription inhibition by DNA lesions *in vitro* (Fig. 3a), we identified newly synthesized RNA transcripts (Fig. 3b) and cDNA products from those transcripts using RT-PCR (Fig. 3c), but we could not detect any PCR products without using reverse transcriptase (Fig. 3c, lanes 11 and 12). As shown in Fig. 3c, the amounts of PCR products (301 bp) from non-irradiated DNA (UV -) were higher than those from UV-irradiated DNA (UV +) until 15 PCR cycles (lanes 5 and 6), indicating that UV-induced DNA lesions blocked RNA synthesis by T7 RNAP. However, after 18 PCR cycles, no difference between RT-PCR products from non-irradiated and UV-irradiated templates could be detected (Fig. 3c, lanes 7–10). These results indicate that RT-PCR using agarose gel electrophoresis for detection (Fig. 3a) might not be suitable for determining the effects of UV-induced DNA damage on transcription because it is necessary to optimize the number of PCR cycles.

**Fig. 4:**
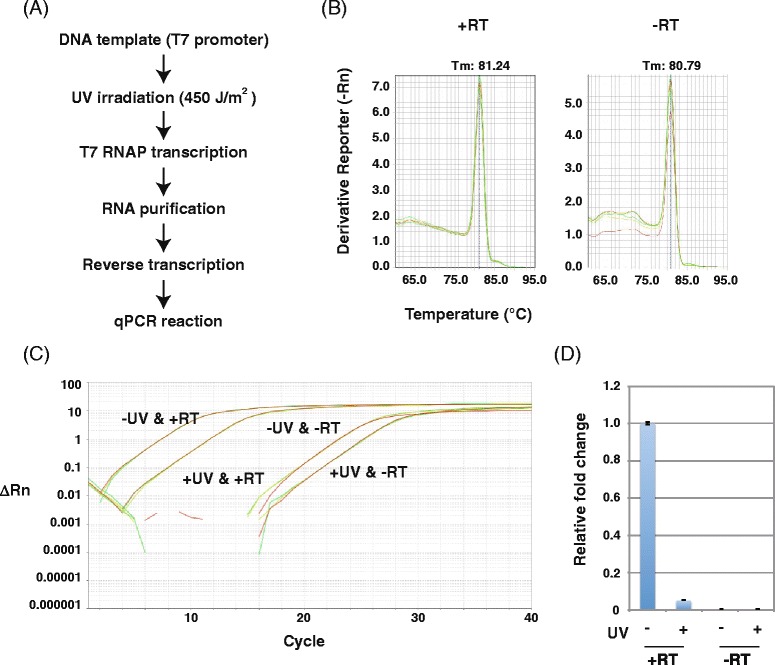
RT-qPCR analysis of transcripts from UV-irradiated DNA templates. **a** Scheme for detecting transcription inhibition from UV-irradiated (450 J/m^2^) DNA templates. **b** Melting curve of RT-qPCR products from RNA transcripts of UV-irradiated DNA templates. Each reaction was run in triplicate. **c** Amplification plot of RT-qPCR analysis of RNA transcripts of UV-irradiated DNA templates. Each reaction was run in triplicate. **d** Relative fold change of transcripts from mock (set as 1.0) and UV-irradiated DNA templates. Data show the mean of three samples ± standard deviation (SD)

**Fig. 5:**
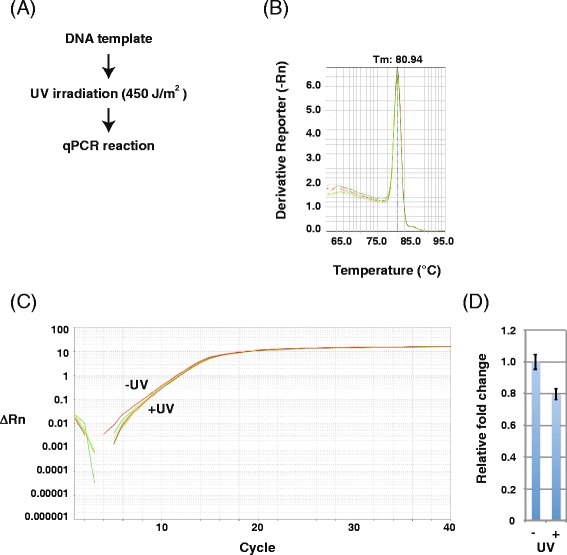
qPCR analysis of UV-irradiated DNA templates. **a** Scheme for detecting DNA damage using qPCR products from UV-irradiated (450 J/m^2^) DNA templates. **b** Melting curve of qPCR products from UV-irradiated DNA templates. Each reaction was run in triplicate. **c** Amplification plot of qPCR analysis of UV-irradiated DNA templates. Each reaction was run in triplicate. **d** Relative fold change of PCR products from mock (set as 1.0) and UV-irradiated DNA templates. Data show the mean of three experiments ± standard deviation (SD)

Therefore, we tested the utility of qPCR for detecting RNA transcription inhibition by UV-induced DNA lesions (Fig. 4a). qPCR is both powerful and sensitive and is used for a broad range of applications. Combined with reverse transcription, it can quantify RNA in cells or tissues. To adapt the new qPCR method, we first assessed primer sets using melting curve analysis (Fig. 4b) and confirmed that these primers generated one PCR product under our experimental conditions. This primer design is a crucial step because inefficient or non-specific primer annealing will negatively affect the quality and reliability of the assay. The amplification plot of the qPCR analysis showed a delay in the accumulation of qPCR products to later cycles, indicating fewer RNA transcripts from UV-irradiated templates (Fig. 4c). Normalized to non-irradiated templates, the amount of RNA transcripts from UV-irradiated templates was markedly decreased by 0.052-fold (Fig. 4d). As expected, qPCR improved detection of the effects of DNA damage on RNA transcription. Without reverse transcriptase, no specific qPCR products were detected (Fig. 4c and d), confirming the origin of these products from T7 RNAP transcription of UV-irradiated DNA templates. Considering the melting curves shown in Fig. 4c and d, however, we cannot rule out the possibility that PCR products derived from DNA remaining after RNA purification. As qPCR proved suitable for detecting damaged DNA templates, we tried to directly apply this method to UV-irradiated templates (Fig. 5). However, while the amplification plot showed slight differences, the method was not sensitive enough to significantly detect damage of UV-irradiated DNA templates (Fig. 5c and d).

**Fig. 6:**
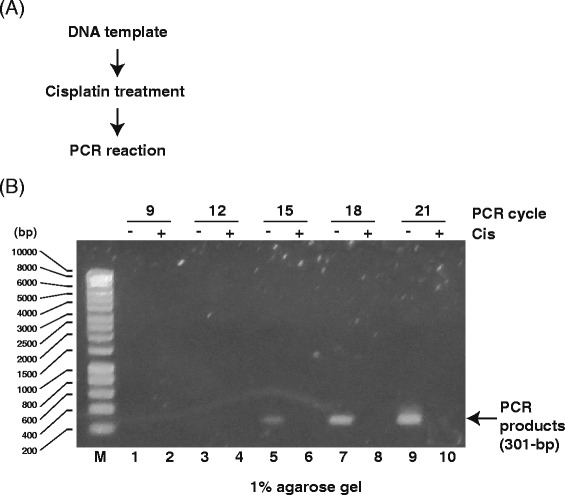
Analysis of PCR products generated from cisplatin-treated DNA templates. **a** Scheme for detecting DNA damage using PCR products from cisplatin-treated DNA templates (drug/nucleotide ratio = 0.005). **b** Agarose gel (1 %) demonstrating PCR products (301 bp) from cisplatin-treated DNA templates. M: size marker. Odd lanes: PCR products from mock DNA templates. Even lanes: PCR products from UV-irradiated DNA templates. DNA was amplified for 9 (*lanes 1 and 2*), 12 (*lanes 3 and 4*), 15 (*lanes 5 and 6*), 18 (*lanes 7 and 8*), or 21 (*lanes 9 and 10*) PCR cycles

**Fig. 7:**
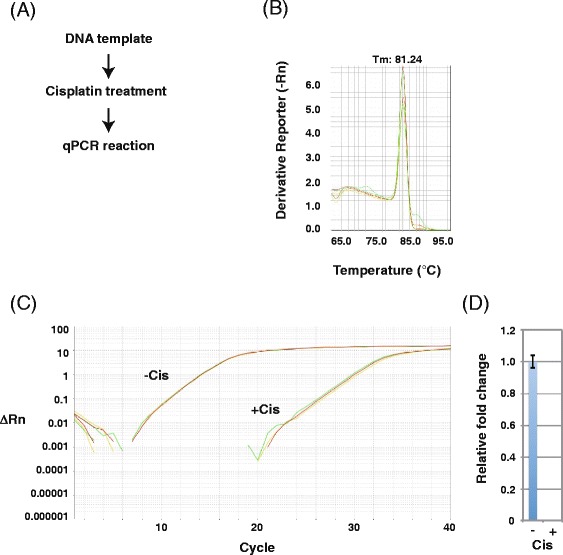
qPCR analysis of cisplatin-treated DNA templates. **a** Scheme for detecting DNA damage using PCR products from cisplatin-treated DNA templates (drug/nucleotide ratio = 0.005). **b** Melting curve of qPCR products from cisplatin-treated DNA templates. Each reaction was run in triplicate. **c** Amplification plot of qPCR analysis of cisplatin-treated DNA templates. Each reaction was run in triplicate. **d** Relative fold change of PCR products from mock (set as 1.0) and cisplatin-treated DNA templates. Data show the mean of three experiments ± standard deviation (SD)

Next, we used cisplatin to directly induce DNA adducts [30, 31] as intrastrand or interstrand crosslinks and monoadducts, which interfere with replication and transcription. Although these adducts are mainly eliminated by NER [32], they are thought to mediate the cytotoxic activity of cisplatin in tumor cells. Using cisplatin-treated DNA samples, we investigated the generation of PCR products from damaged templates. Unlike with UV-damaged templates, conventional PCR (Fig. 6a) revealed differences between cisplatin-treated and untreated DNA (Fig. 6b). These results suggest that cisplatin DNA adducts efficiently block DNA synthesis by Taq DNA polymerase and/or prevent primer annealing. Consistent with this observation, we obtained similar results using qPCR to detect cisplatin-induced DNA adducts, revealing a clear difference between damaged and non-damaged DNA (Fig. 7).

**Fig. 8:**
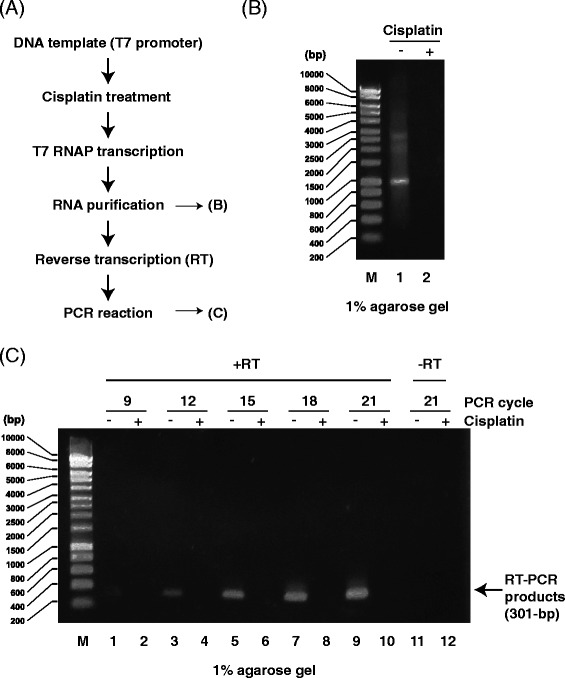
Analysis of PCR products generated by RT-PCR from RNA transcripts of cisplatin-treated DNA templates. **a** Scheme for detecting transcription inhibition using RNA transcripts from cisplatin-treated DNA templates (drug/nucleotide ratio = 0.005). **b** Agarose gel (1 %) demonstrating RNA transcripts from cisplatin-treated DNA templates. M: size marker. Lane 1: RNA transcripts from mock DNA templates. Lane 2: RNA transcripts from cisplatin-treated DNA templates. **c** Agarose gel (1 %) demonstrating RT-PCR products (301 bp) from cisplatin-treated DNA templates. M: size marker. Odd lanes: RT-PCR products from mock DNA templates. Even lanes: RT-PCR products from cisplatin-treated DNA templates. DNA was amplified for 9 (*lanes 1 and 2*), 12 (*lanes 3 and 4*), 15 (*lanes 5 and 6*), 18 (*lanes 7 and 8*), or 21 (*lanes 9 and 10*) PCR cycles. Lanes 11 and 12: no RT reaction

**Fig. 9:**
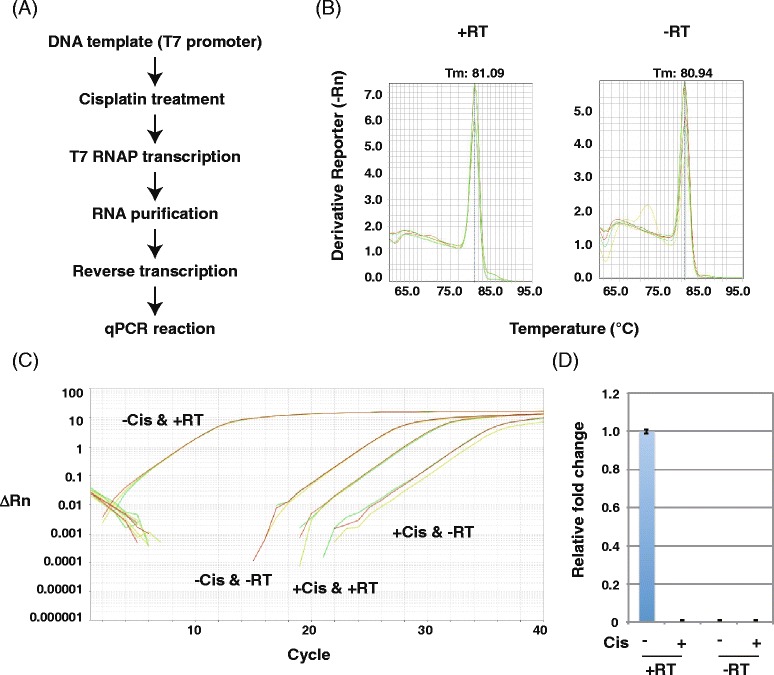
RT-qPCR analysis of transcripts from cisplatin-treated DNA templates. **a** Scheme for detecting transcription inhibition from cisplatin-treated DNA templates (drug/nucleotide ratio = 0.005). **b** Melting curve of RT-qPCR products from RNA transcripts of cisplatin-treated DNA templates. Each reaction was run in triplicate. **c** Amplification plot of RT-qPCR analysis of RNA transcripts of cisplatin-treated DNA templates. Each reaction was run in triplicate. **d** Relative fold change of transcripts from mock (set as 1.0) and cisplatin-treated DNA templates. Data show the mean of three samples ± standard deviation (SD)

Previous biochemical studies demonstrated that T7 RNAP stalls at cisplatin DNA adducts [33, 34]. Accordingly, when we tested the effects of cisplatin on DNA templates via transcription and RT-PCR (Fig. 8a), we detected newly synthesized RNA transcripts from untreated DNA templates (Fig. 8b, lane 1) but not from cisplatin-treated templates (Fig. 8b, lane 2) and detected cDNA from untreated DNA templates (Fig. 8c, lanes 3, 5, 7, and 9) but not cisplatin-treated templates (Fig. 8c, lanes 4, 6, 8, and 10). No PCR products were detected in the absence of RT (Fig. 8c, lanes 11 and 12). These results indicate that cisplatin DNA adducts inhibited T7 RNAP transcription initiation and/or elongation. qPCR melting curve analysis (Fig. 9a) indicated that the primer set generated one PCR product after RNA transcription from cisplatin-treated DNA templates (Fig. 9b), suggesting that the PCR product is specific. The amplification plot (Fig. 9c) and the relative fold change (Fig. 9d) showed little T7 RNAP transcription of cisplatin-treated DNA templates, and insignificant amounts of PCR products were generated without RT, indicating that this method can detect the inhibition of RNA synthesis by cisplatin DNA damage.

## Conclusions

In conclusion, conventional PCR and qPCR in the absence of T7 RNAP transcription can detect chemically induced DNA lesions such as cisplatin DNA adducts but not UV-induced lesions. Only the combination of T7 RNAP transcription and qPCR can detect both cisplatin- and UV-induced DNA lesions that interfere with transcription. Therefore, our results support the idea analysis of transcription products can be used to detect damage in DNA templates, consistent with the model of TCR [7, 25]. Our new method might reveal DNA lesions that cannot be detected by conventional replication-based methods and should facilitate research on DNA damage responses.

## Abbreviations

4NQO: 4-nitroquinoline 1-oxide
6-4 pp: 6–4 photoproduct
CPD: Cyclobutane pyrimidine dimer
NER: Nucleotide excision repair
PCR: Polymerase chain reaction
qPCR: Real-time quantitative PCR
RT: Reverse transcription
T7 RNAP: T7 RNA polymerase
TCR: Transcription-coupled repair
UV: Ultraviolet light
